# Neural decoding of single vowels during covert articulation using electrocorticography

**DOI:** 10.3389/fnhum.2014.00125

**Published:** 2014-03-07

**Authors:** Shigeyuki Ikeda, Tomohiro Shibata, Naoki Nakano, Rieko Okada, Naohiro Tsuyuguchi, Kazushi Ikeda, Amami Kato

**Affiliations:** ^1^Graduate School of Information Science, Nara Institute of Science and TechnologyIkoma, Japan; ^2^Graduate School of Life Science and Systems Engineering, Kyushu Institute of TechnologyKitakyushu, Japan; ^3^Department of Neurosurgery, Kinki University Faculty of MedicineSayama, Japan; ^4^Department of Neurosurgery, Graduate School of Medicine, Osaka City UniversityOsaka, Japan; ^5^Core Research for Evolutionary Science and Technology, Japan Science and Technology AgencyKawaguchi, Japan

**Keywords:** covert articulation, single vowel, neural decoding, electrocorticography (ECoG), functional mapping

## Abstract

The human brain has important abilities for manipulating phonemes, the basic building blocks of speech; these abilities represent phonological processing. Previous studies have shown change in the activation levels of broad cortical areas such as the premotor cortex, the inferior frontal gyrus, and the superior temporal gyrus during phonological processing. However, whether these areas actually convey signals to representations related to individual phonemes remains unclear. This study focused on single vowels and investigated cortical areas important for representing single vowels using electrocorticography (ECoG) during covert articulation. To identify such cortical areas, we used a neural decoding approach in which machine learning models identify vowels. A decoding model was trained on the ECoG signals from individual electrodes placed on the subjects' cortices. We then statistically evaluated whether each decoding model showed accurate identification of vowels, and we found cortical areas such as the premotor cortex and the superior temporal gyrus. These cortical areas were consistent with previous findings. On the other hand, no electrodes over Broca's area showed significant decoding accuracies. This was inconsistent with findings from a previous study showing that vowels within the phonemic sequence of words can be decoded using ECoG signals from Broca's area. Our results therefore suggest that Broca's area is involved in the processing of vowels within phonemic sequences, but not in the processing of single vowels.

## 1. Introduction

Language appeared during the course of human evolution, enabling us to communicate with others. In oral communication, speech consists of complicated sounds which are rarely found in other animals. Phonemes are the basic building blocks that make up speech. The human brain can flexibly manipulate phonemes to compose and decompose syllables or words, in what is known as phonological processing (McBride-Chang, 1996). Previous studies have reported that broad cortical areas such as the premotor cortex, the inferior frontal gyrus (IFG), and the superior temporal gyrus (STG) show changes in activation levels during phonological processing (Vigneau et al., 2006). However, the question remains as to whether these areas actually convey signals on the phoneme-related representations needed for manipulating phonemes during phonological processing.

Decoding-based approaches provide sophisticated methods for identifying cognitive or perceptual states from brain activity (Haynes and Rees, 2006). Recent studies have demonstrated that phoneme-related representations can be extracted from brain activity during covert speech, which is assumed to include all language processes other than the motor execution stage. Some studies have reported successful identification of vowels using electroencephalography (EEG) (DaSalla et al., 2009) or electrocorticography (ECoG) (Leuthardt et al., 2011). However, those studies did not localize cortical areas for the identification of vowels. Pei et al. (2011) reported that phonemes within words could be identified using ECoG signals measured during covert speech of those words, and they localized cortical areas carrying information for the identification of phonemes. They decoded vowels within the phonemic sequence of words using ECoG signals. However, neural substrates for the representation of phonemes may differ when a vowel is processed within a phonemic sequence and when it is processed alone. Addressing this issue contributes to the understanding of brain function which manipulates basic speech sounds.

To this end, we measured ECoG signals while subjects covertly articulated single vowels. Based on decoding accuracies using these ECoG signals, cortical areas in which brain activity discriminated single vowels were identified. Cortical areas such as the premotor cortex and STG were identified; these cortical areas matched previous findings (Pei et al., 2011). STG is involved in covert speech production, which is evident in that cortical activations increased over STG during covert word production (Pei et al., 2010). The premotor cortex is assumed to be important for articulatory planning (Duffau et al., 2003), and pronounced activations over the premotor cortex were found during covert word production (Pei et al., 2010). In addition, previous findings revealed anatomical connectivity between the premotor cortex and STG (Saur et al., 2008); the connections are involved in a dorsal stream which is important for mapping sound to articulation. In contrast to previous findings (Pei et al., 2011), no electrodes over Broca's area showed significant decoding accuracies. This was considered to be because Broca's area is assumed to be important for the segmentation of words into individual phonemes as a part of phonological processing (Zatorre et al., 1992, 1996; Burton et al., 2000). Our results suggest that Broca's area is involved in the processing of vowels within phonemic sequences, but not in the processing of single vowels.

## 2. Materials and methods

### 2.1. Subjects

Four subjects with intractable epilepsy (one male, three females) participated in our investigation. The subjects underwent temporary placement of subdural electrodes to localize seizure foci before surgical treatment of their epilepsy. All subjects provided written informed consent to participate in this study. All study protocols were approved by the ethics committees of both Kinki University Faculty of Medicine (21–135) and Nara Institute of Science and Technology (2203).

**Figure 1:**
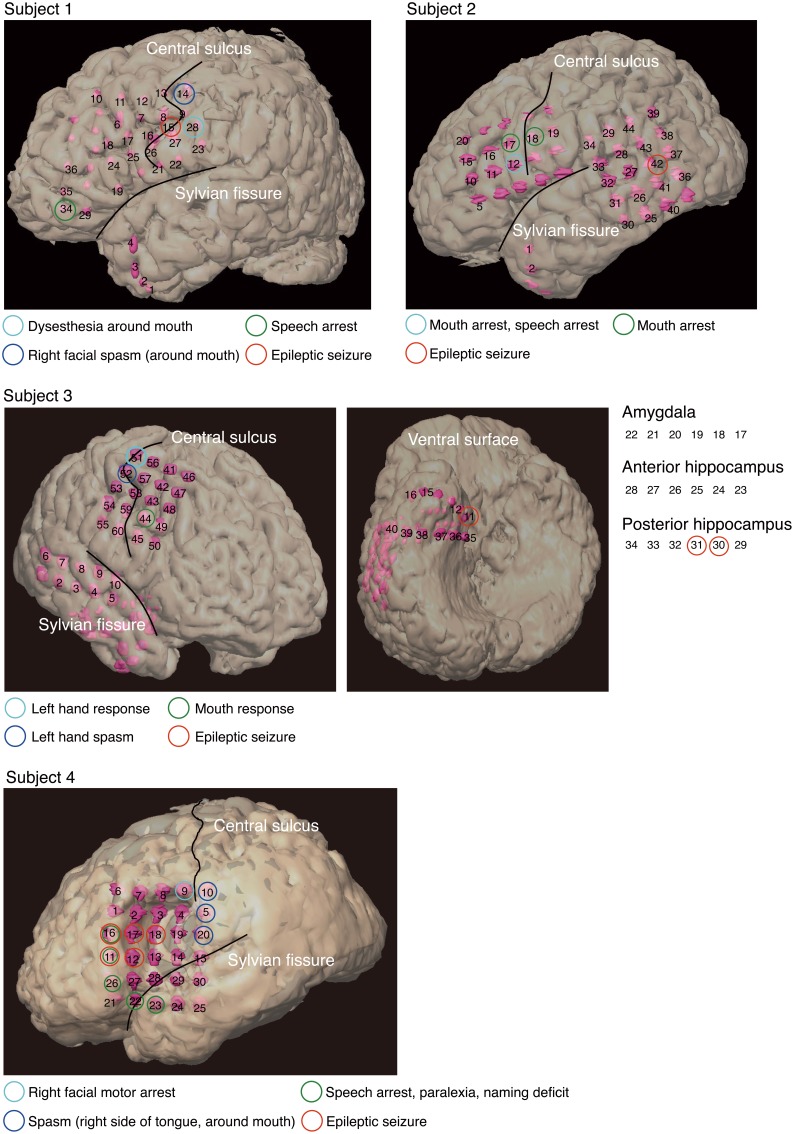
**Electrode locations and responses to electrical stimulation for each subject, superimposed onto positions of the implanted subdural electrodes**. The two black lines delineate the locations of the central sulcus and Sylvian fissure. Pink objects represent the electrodes. Unnumbered electrodes are those that were not used in this analysis due to severe measurement noise. Each colored circle indicates a response to electrical stimulation at the corresponding electrode location. Subject 3 had electrodes (11–40) over the right ventral temporal lobe. To facilitate visualization, electrodes 17–34 are not superimposed on the ventral surface shown in the figure. Electrodes 17–22 are shown over the amygdala, electrodes 23–28 over the anterior hippocampus, and electrodes 29–34 over the posterior hippocampus.

Individual subjects had subdural electrodes placed over the frontal, parietal, and temporal regions (Figure 1). Only Subject 3 had electrodes placed on the amygdala and anterior and posterior hippocampus. Electrodes on all subjects except Subject 3 were localized over the left hemisphere, and electrodes on all subjects were based solely on the requirements of clinical evaluations, without any alterations made for endpoints of this study.

We conducted a Wada test for subjects to specify the hemispheric dominance for language (Wada and Rasmussen, 1960). As shown in Table 1, the left hemisphere was dominant in Subjects 1 and 3, and both hemispheres were dominant in Subjects 2 and 4.

**Table 1:** **Clinical profiles of subjects**.

**Subject**	**Age**	**Sex**	**Language dominance**	**Electrode location**
1	17	M	Left	Left frontal-parietal-temporal
2	38	F	Right and left	Left frontal-parietal-temporal
3	34	F	Left	Right frontal-parietal-temporal-amygdala-hippocampus
4	22	F	Right and left	Left frontal-parietal-temporal

Because we postoperatively preserved function in cortical areas for each subject, we performed electrical stimulation mapping (ESM), which enabled us to localize epileptic foci for each subject and to identify critical sensory, motor or language areas. Sites with positive findings by electrical stimulation are shown in Table 2.

**Table 2:** **Sites with positive findings identified via electrical stimulation**.

	**Electrode number**	**Responses**
Subject 1	14	Right facial spasm (around mouth)
	15	Epileptic seizure
	28	Dysesthesia around mouth
	34	Speech arrest
Subject 2	12	Mouth arrest, speech arrest
	17, 18	Mouth arrest
	42	Epileptic seizure
Subject 3	11, 30, 31	Epileptic seizure
	44	Mouth response
	51	Left hand response
	52	Left hand spasm
Subject 4	5, 10, 20	Spasm (right side of tongue, around mouth)
	9	Right facial motor arrest
	11, 12, 16, 17, 18	Epileptic seizure
	11, 16, 22, 23, 26	Speech arrest, paralexia, naming deficit

In three of the four subjects, we found electrodes in which electrical stimulation caused a disruption of speech (Speech arrest) (Table 2). We defined the cortical areas showing “Speech arrest” as Broca's area. Note that the cortical area on which electrode 12 in Subject 2 was placed was assumed not to be Broca's area because the cortical area was close to the central sulcus. In addition, because electrode 23 in Subject 4 was placed on the temporal part, this area was not Broca's area.

### 2.2. Experimental setup

**Figure 2:**
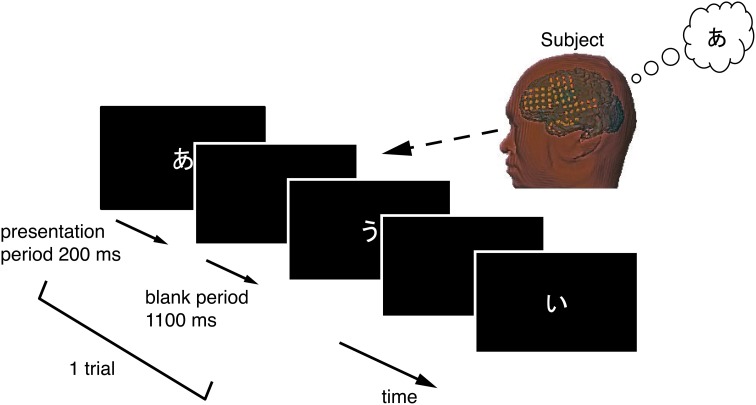
**Temporal sequence in the vowel-articulating task**. The solid arrow indicates the time-line. Each trial consisted of a presentation period followed by a blank period. The subject covertly articulated the vowel one single time during the blank period.

Each subject performed a task in this study while sitting upright in a hospital bed. Figure 2 illustrates the task procedure. Each trial consisted of a presentation and a blank period. The presentation period lasted 200 ms, and the blank period lasted 1100 ms, so each trial lasted 1300 ms overall. We selected three of the five Japanese vowels because the task was designed to be as brief as possible, in accordance with the medical judgment of the doctors collaborating in this study. A task consisted of 90 trials, with 30 for each vowel. Each task thus lasted about 120 s. Each vowel was shown on a liquid crystal display monitor during the presentation period, and the subjects were asked to covertly articulate the vowel one single time during the blank period without intentional movements such as of the lips or tongue. The three vowels selected were /a/, /i/, and /u/ (/a/:/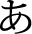/, /i/:/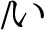/, and /u/:/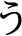/) Each was presented as a white hiragana letter against a black background. The order of presentation was randomized so that subjects were unable to predict which vowel would be presented in a given trial.

### 2.3. Data collection

UZN-series electrodes (Unique Medical Corporation, Tokyo, Japan) were used, and signals from the electrodes were recorded using an EEG1000-series measurement system (Nihon Kohden Corporation, Tokyo, Japan). Intra-electrode distance was 10 mm, and the sampling frequency for analog-to-digital conversion was 1000 Hz. All electrodes were referenced to a scalp electrode placed on the nasion. In all subjects, electrodes containing severe measurement noise were removed from the analysis. These electrodes are shown as unnumbered electrodes (Figure 1). All data were analyzed using Matlab 2011a software (The MathWorks, Natick, MA, USA).

### 2.4. Feature extraction

**Figure 3:**
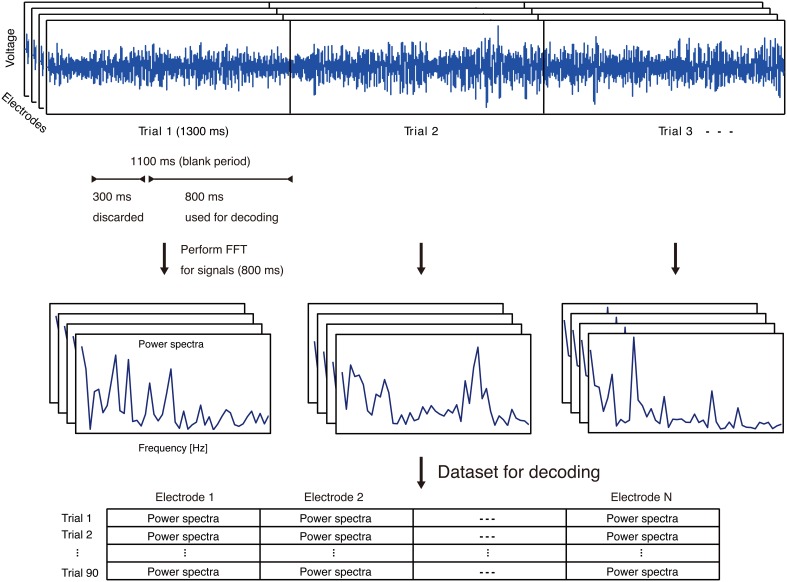
**Feature extraction process using ECoG signals**. **Top:** Representative ECoG signals recorded in individual trials. The ECoG signals recorded in an early stage of individual trials could reflect immediate responses to visual stimuli. In order to decode single vowels based on information from covert articulation, and not information from the visual stimuli, we used ECoG signals arriving only after 300 ms into the blank period. **Middle:** Representative power spectra extracted from each trial and each electrode. **Bottom:** For each trial, power spectra from all electrodes. N is the number of electrodes on an individual subject.

The ECoG signals recorded in an early stage of individual trials could reflect immediate responses to visual stimuli. In order to decode single vowels based on information from covert articulation, and not information from the visual stimuli, we used ECoG signals arriving only after 300 ms into the blank period. (Figure 3).

For ECoG features as input to decoding analyses, we focused on the high-gamma frequency band of ECoG signals (70–110 Hz). The high-gamma band is assumed to be associated with auditory perception of phonemes or word production (Crone et al., 2001a,b; Canolty et al., 2007; Pei et al., 2010). For each electrode, the power spectra in the high-gamma bands were extracted from ECoG signals in the blank period of individual trials using fast Fourier transform (FFT) (Figure 3). With selection of the high-gamma band, we avoided the 60-Hz power noise and associated harmonics (e.g. 120 Hz). The power spectra were used as feature vectors for decoding analyses.

For normalization of power spectra, we divided all trials into testing trials and training trials, and then calculated z-scores from each power spectrum across the training trials for individual electrodes. To normalize testing trials without using those trials, we calculated z-scores from each power spectrum across the testing trials using the mean and standard deviation calculated from each power spectrum across the training trials.

### 2.5. Evaluation

We constructed a linear classifier (decoder) to classify vowels from feature vectors on a trial-by-trial basis. The decoder calculated the linearly weighted sum of the features plus a bias for each class (/a/, /i/, and /u/), and the class with the maximum value was chosen as the classified class. Individual weights and biases were determined using a support vector machine (SVM) with the linear kernel applied to the training trials (Vapnik, 1998). SVM is a commonly used algorithm in the field of brain reading (Haynes and Rees, 2006). We used LIBSVM (Chang and Lin, 2011) in Matlab to implement the SVM.

To evaluate decoding accuracy, we performed a cross-validation approach using all of the trials. In this approach, all of the trials were divided into 15 subgroups; 14 of the subgroups were used for training a classifier, and the remaining subgroup was used for testing the classifier. This procedure was repeated 15 times, using all of the trials of all of the subgroups as testing trials once (15-fold cross-validation). Decoding accuracy was calculated as a percentage of the correct classifications.

For each subject, we computed the decoding accuracy from each electrode, and derived a *p*-value corresponding to each decoding accuracy from a distribution given by the normal approximation to the binomial distribution. The mean of the distribution is *nc* (*n*: total number of trials, *c*: chance level 0.33); standard deviation is calculated according to $\sqrt{nc(1-c)}$. We then determined cortical representation areas of vowels in covert articulation based on decoding accuracies significantly greater (*p* < 0.05) than the level of chance.

## 3. Results

**Figure 4:**
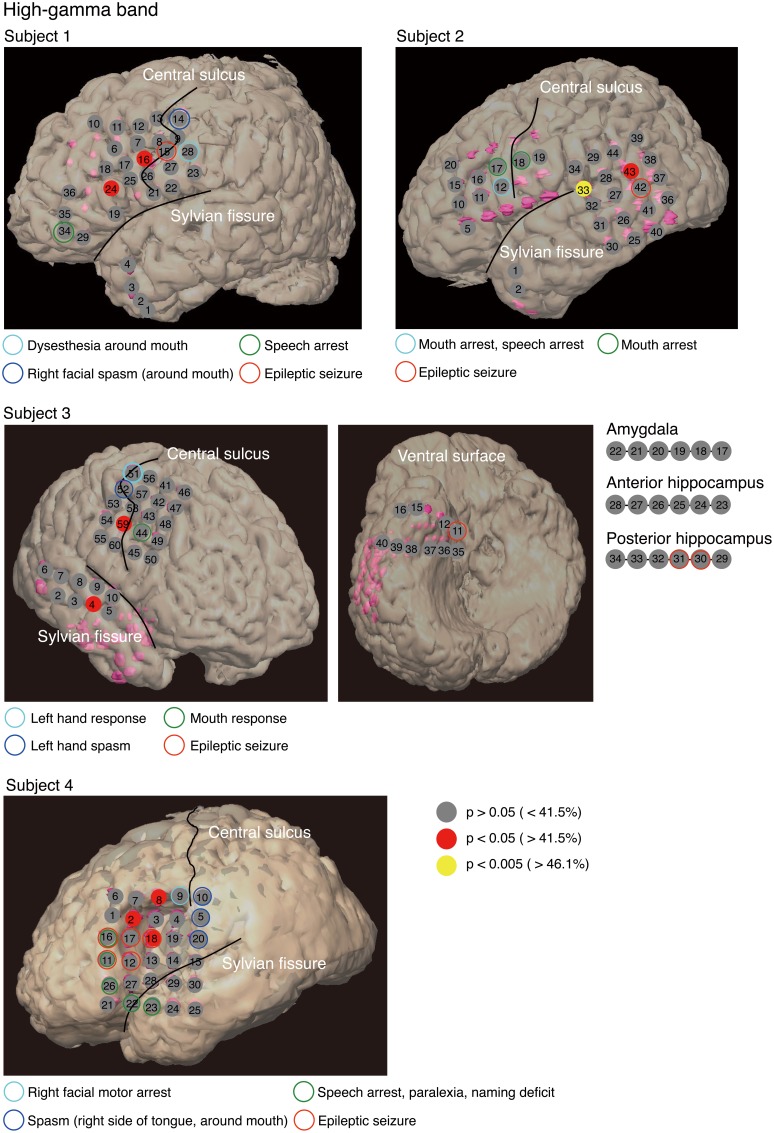
**Decoding accuracies acquired from the high-gamma power spectra for each electrode in individual subjects**. We estimated the decoding accuracy of each electrode by 15-fold cross-validation. Decoding accuracies were superimposed onto the electrode map of each subject. To facilitate visualization, for Subject 3, electrodes 17–34 are not superimposed onto the ventral surface shown in the figure. The color filling each circle represents the level of significance of the decoding accuracy; *p*-values corresponding to decoding accuracy were computed from a distribution given by the normal approximation to the binomial distribution (mean: *nc*, standard deviation: $\sqrt{nc(1-c)}$, *n*: total number of trials, *c*: chance level 0.33).

To identify cortical areas in which brain activity discriminates single vowels of covert articulation, we statistically evaluated decoding accuracies from individual electrodes in each subject. Decoding accuracies corresponding to the electrodes were superimposed onto the electrode map of each subject (Figure 4). In addition, Table 3 showed over which cortical areas statistically significant electrodes were placed. The results shown in Table 3 demonstrated that the cortical areas able to discriminate single vowels were the primary motor area, the premotor cortex, STG, and so on. Decoding accuracies from these areas ranged from 42.2 to 46.7% (chance level 33.3%). Furthermore, we found that no electrodes over Broca's area showed significant decoding accuracies (Table 4).

**Table 3:** **Cortical areas showing significant decoding accuracies in use of the high-gamma power spectra**.

	**Electrode number**	**Cortical area**	**Accuracy (%) (*p*)**
Subject 1	16	Left primary motor area	42.2 (*p* < 0.05)
	24	Left premotor cortex	42.2 (*p* < 0.05)
Subject 2	33	Left superior temporal gyrus	46.7 (*p* < 0.005)
	43	Left angular gyrus	42.2 (*p* < 0.05)
Subject 3	4	Right superior temporal gyrus	44.4 (*p* < 0.05)
	59	Right primary somatosensory area	42.2 (*p* < 0.05)
Subject 4	2	Left dorsolateral prefrontal cortex	42.2 (*p* < 0.05)
	8	Left premotor cortex	42.2 (*p* < 0.05)
	18	Left premotor cortex	42.2 (*p* < 0.05)

**Table 4:** **Decoding accuracies in Broca's area using the high-gamma power spectra**.

	**Electrode number**	**Accuracy (%)**	***p*-value**
Subject 1	34	33.3	0.5
Subject 2	NA		
Subject 3	NA		
Subject 4	11	32.2	0.59
	16	40.0	0.09
	22	25.6	0.94
	26	30.0	0.75

## 4. Discussion

Our purpose was to identify cortical areas in which brain activity can discriminate single vowels of covert articulation. We identified cortical areas such as the premotor cortex and STG (Figure 4); these cortical areas were consistent with the previous findings (Pei et al., 2011). We also found that no electrodes over Broca's area showed significant decoding accuracies (Table 4), whereas Pei et al. (2011) reported that ECoG signals from the area carry information about the discrimination of vowels within phonemic sequences. This difference could be attributed to the function of Broca's area. When a subject covertly articulates a word, the word is segmented in this area into individual phonemes as a part of phonological processing (Zatorre et al., 1992, 1996; Burton et al., 2000). In addition, the high-gamma power increased over Broca's area during segmentation of words (Herman et al., 2013). Therefore, in the previous study (Pei et al., 2011), individual vowels within phonemic sequences may have been decoded using the high-gamma band from Broca's area, which contains information about individual vowels segmented from words. Subjects in our study covertly articulated only single vowels, which thus did not require segmentation by Broca's area. This is why single vowels could not be decoded using the high-gamma band from Broca's area.

Since previous findings reported that cortical activations in the theta (4–7 Hz), alpha (8–13 Hz), beta (14–30 Hz) bands are associated with language processing (Bastiaansen and Hagoort, 2006; Giraud and Poeppel, 2012), we investigated cortical areas important for representation of single vowels when using these bands for decoding. Table 5 showed over which cortical areas statistically significant electrodes were placed. We found significant decoding accuracies in Broca's area when using the alpha or beta power spectra. This was expected because a previous study suggested that Broca's area has a motoric function which translates speech into articulatory code (Hickok and Poeppel, 2004). Furthermore, event-related desynchronization (ERD) occurred in alpha and beta bands over Broca's area during silent reading of words (Goto et al., 2011). Because ERD is assumed to include motor-related information (Crone et al., 1998), ECoG signals in the alpha and beta bands over Broca's area may contain information about the articulatory code of single vowels. Therefore, our results suggest that when subjects covertly articulate a single vowel, ECoG signals over Broca's area contain information about covert articulation of a single vowel, but not about segmentation of a phoneme sequence.

**Table 5:** **Cortical areas showing significant decoding accuracies in use of the theta, alpha, and beta power spectra**.

**Frequency band**	**Subject number**	**Electrode number**	**Cortical area**	**Accuracy (%) (***p***)**
Theta	1	2	Left anterior temporal cortex	42.2 (*p* < 0.05)
	2	NA		
	3	NA		
	4	NA		
Alpha	1	34	Broca's area	42.2 (*p* < 0.05)
	2	NA		
	3	NA		
	4	26	Broca's area	42.2 (*p* < 0.05)
Beta	1	3	Left anterior temporal cortex	42.2 (*p* < 0.05)
		35	Left inferior frontal gyrus	42.2 (*p* < 0.05)
	2	32	Left superior temporal gyrus	44.4 (*p* < 0.05)
		37	Left angular gyrus	43.3 (*p* < 0.05)
	3	17	Right amygdala	47.8 (*p* < 0.005)
		26	Right anterior hippocampus	50.0 (*p* < 0.005)
		32	Right posterior hippocampus	44.4 (*p* < 0.05)
	4	25	Left superior temporal gyrus	45.6 (*p* < 0.01)
		26	Broca's area	55.6 (*p* < 0.005)
		27	Left inferior frontal gyrus	43.3 (*p* < 0.05)

For the high-gamma band (Figure 4), significant decoding accuracies were seen in the primary motor area. We speculated that these results are associated with motor imagery. Motor imagery is usually performed unconsciously during movement preparation (Lotze and Halsband, 2006). Various studies, such as (Sharma et al., 2008; Miller et al., 2010), have reported that the primary motor area is activated during motor imagery tasks. In addition, (Wildgruber et al., 1996) suggested that covert speech is associated with motor imagery. In contrast, other studies have found that the primary motor cortex is barely activated during covert speech (Palmer et al., 2001; Huang et al., 2002), and also that individual vowels within words cannot be decoded using ECoG signals from the area (Pei et al., 2011). Given that words used in the previous study consisted of more than three phonemes, movement preparation for covert articulation of a word is assumed to be more complicated than for that of a single vowel. ECoG signals during covert articulation of a word thus carry information about complicated movement preparation. The present results suggest that decoding individual vowels within words using ECoG signals may be more difficult than decoding single vowels using ECoG signals during covert articulation of single vowels.

Some of the other areas important for the representation of single vowels were consistent with previous findings (Pei et al., 2011); these areas were the premotor cortex and STG (Table 3). The premotor cortex is assumed to be associated with motor planning (Duffau et al., 2003) and is activated not only during overt speech, but also during covert speech (Price, 2012). Single vowels may plausibly be decodable using ECoG signals from the premotor cortex. Regarding STG, some studies have provided evidence that this area is important for representing phonetic contents (Obleser et al., 2006; Chang et al., 2010; Leuthardt et al., 2011). This area might therefore represent auditory images of individual vowels while subjects are covertly articulating single vowels. In addition, previous findings revealed anatomical connectivity between the premotor cortex and STG (Saur et al., 2008); the connections are involved in a dorsal stream which is important for mapping sound to articulation.

## 5. Limitations

The subjects in this study were intractable epilepsy patients with a limited amount of time to participate in the experiment. Therefore, our experimental design did not include control conditions (i.e. using the same vowels but without covert speech and using non-language stimuli) to extract ECoG signals about covert articulation and vowel perception. Note that since we used ECoG signals arriving only after 300 ms into the blank period as input for decoding analyses, input used for decoding is assumed not to contain information from visual stimuli. This suggests that our ability to decode single vowels was based on information of neural processes involved in covert articulation, rather than information of processes involved in visual stimuli.

In this study, we concluded that Broca's area is involved in the processing of vowels within phonemic sequences, but not in processing single vowels. To confirm our conclusion, we should investigate whether vowels within the phonemic sequence of words can be decoded using ECoG signals from Broca's area. Due to their time constraints, subjects in this study were unable to perform the task of covert articulation of vowels within phonemic sequences.

### Conflict of interest statement

The authors declare that the research was conducted in the absence of any commercial or financial relationships that could be construed as a potential conflict of interest.
